# Superconductivity at Pd/Bi_2_Se_3_ Interfaces Due to Self-Formed PdBiSe Interlayers

**DOI:** 10.3390/ma17225460

**Published:** 2024-11-08

**Authors:** Kaixuan Fan, Ze Hua, Siyao Gu, Peng Zhu, Guangtong Liu, Hechen Ren, Ruiwen Shao, Zhiwei Wang, Li Lu, Fan Yang

**Affiliations:** 1 Center for Joint Quantum Studies and Department of Physics, School of Science, Tianjin University, Tianjin 300354, China; fankaixuan@tju.edu.cn (K.F.); gusiyao@tju.edu.cn (S.G.); ren@tju.edu.cn (H.R.); 2 Tianjin Key Laboratory of Low Dimensional Materials Physics and Preparing Technology, Department of Physics, Tianjin University, Tianjin 300354, China; 3 Beijing National Laboratory for Condensed Matter Physics, Institute of Physics, Chinese Academy of Sciences, Beijing 100190, China; gtliu@iphy.ac.cn (G.L.); lilu@iphy.ac.cn (L.L.); 4 Beijing Advanced Innovation Center for Intelligent Robots and Systems, School of Medical Technology, Beijing Institute of Technology, Beijing 100081, China; huaze@bit.edu.cn; 5 Centre for Quantum Physics, Key Laboratory of Advanced Optoelectronic Quantum Architecture and Measurement (MOE), School of Physics, Beijing Institute of Technology, Beijing 100081, China; 3120215761@bit.edu.cn

**Keywords:** Bi_2_Se_3_, topological insulator, topological superconductivity

## Abstract

Understanding the physical and chemical processes at the interfaces of metals and topological insulators is crucial for the development of the next generation of topological quantum devices. Here, we report the discovery of robust superconductivity in Pd/Bi_2_Se_3_ bilayers fabricated by sputtering Pd on the surface of Bi_2_Se_3_. Through transmission electron microscopy measurements, we identify that the observed interfacial superconductivity originates from the diffusion of Pd into Bi_2_Se_3_. In the diffusion region, Pd chemically reacts with Bi_2_Se_3_ and forms a layer of PdBiSe, a known superconductor with a bulk transition temperature of 1.5 K. Our work provides a method for the introduction of superconductivity into Bi_2_Se_3_, laying the foundation for the development of sophisticated Bi_2_Se_3_-based topological devices.

## 1. Introduction

The non-trivial band topology of three-dimensional topological insulators (3D TIs) [1,2,3] makes them an advantageous platform for the development of various types of topological devices [4]. For instance, a spinless topological superconductor can be artificially realized by introducing superconductivity via proximity effects into electronic states where spin degeneracy is lifted, such as the topological surface states of 3D TIs [5,6]. Such spinless superconductors are predicted to host Majorana fermions at their boundaries. When confined to zero dimensions, Majorana fermions develop into Majorana zero modes that obey non-Abelian statistics. Such quasiparticles are theoretically proposed as building blocks for fault-tolerant quantum computing [7,8,9,10]. Furthermore, the unique helical spin texture of topological surface states also renders 3D TIs promising for spintronic applications [10,11,12,13].

A deep understanding of the physical and chemical processes at metal/TI interfaces is essential for the design and fabrication of TI-based devices. Different functional interfaces can be achieved via the appropriate selection of metals and precise control of the deposition conditions. Previously, it was reported that the diffusion of sputtered Pd into the 3D TI (Bi_1−x_Sb_x_)_2_Te_3_ led to the self-formation of a superconducting PdTe_2_ layer at the interface [14]. Similarly, signatures of superconductivity were also reported in annealed Pd/Bi_2_Se_3_ bilayer structures, but the origin of the superconductivity was unclear [15]. In this article, we report the observation of robust superconductivity in Pd/Bi_2_Se_3_ bilayers fabricated by sputtering Pd onto Bi_2_Se_3_. Although both Pd and Bi_2_Se_3_ are non-superconducting materials, the Pd/Bi_2_Se_3_ bilayer was found to exhibit a sharp superconducting transition at $T_{c}\approx$ 1.2 K. Through atomically resolved structural analysis with transmission electron microscopy (TEM), it was determined that the observed superconductivity arose from a superconducting PdBiSe layer forming at the Pd/Bi_2_Se_3_ interface. Our work offers a new approach for the introduction of superconductivity into Bi_2_Se_3_ and paves the way for the development of Bi_2_Se_3_-based hybrid superconducting devices. 

## 2. Materials and Methods

### 2.1. Crystal Growth

High-quality single crystals of Bi_2_Se_3_ were grown using the melt method. Stoichiometric mixtures of Bi (99.9999% purity) and Se (99.999% purity) elements were melted in an evacuated quartz tube and then slowly cooled down to 550 °C over 50 h. After this, the crystals were kept in the quartz tube at 550 °C for 3 days and then cooled down to room temperature. Finally, large Bi_2_Se_3_ single crystals with shiny surfaces were obtained.

### 2.2. Device Fabrication

Bi_2_Se_3_ flakes were mechanically exfoliated from bulk single crystals and then transferred onto SiO_2_ (300 nm)/Si substrates using polyethylene tape, which produces less residual glue compared to the commonly used Scotch tape. Flakes with regular shapes and a suitable thickness were selected for device fabrication. The resist pattern of Pd electrodes were prepared in a single step of electron-beam lithography (EBL) using an eLINE EBL system manufactured by Raith (Dortmund, Germany). To remove possible residual resist and native oxide, the contact areas were gently etched in Ar plasma for 40 s before the deposition of Pd. The etching power and Ar pressure were 2.6 W and 0.1 Pa, respectively. After etching, about 100 nm of Pd was deposited by magnetron sputtering with power of 100 W and an argon pressure of 0.7 Pa. The lift-off of Pd was performed in acetone at 60 °C. 

**Figure 1:**
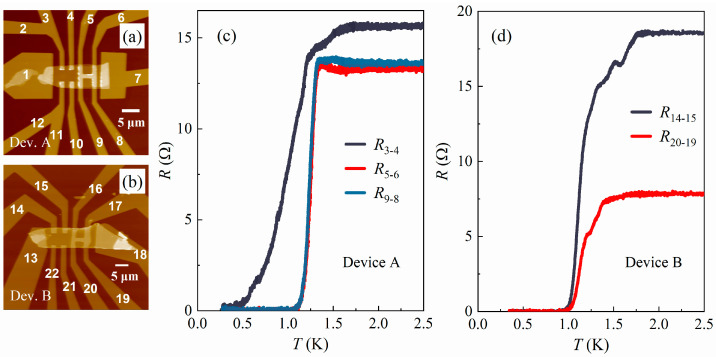
(**a**,**b**) AFM images of devices A and B, with electrode numbers indicated. The thickness of the Bi_2_Se_3_ flakes in devices A and B was measured to be 73 nm and 66 nm, respectively. (**c**,**d**) The *R*(*T*) curves of devices A and B, measured with an excitation current of 50 nA.

After fabrication, the devices were characterized using an atomic force microscope (AFM) manufactured by Being Nano-Instruments (Guangzhou, China). In this paper, we present the data from two independent devices, labeled as devices A and B, respectively. The AFM images of these devices are shown in Figure 1a,b, with all electrodes labeled accordingly.

### 2.3. Transport Measurements

Electron transport measurements were performed in a ^3^He cryostat with a base temperature of 270 mK in magnetic fields up to 14 T. The resistance was measured in a four-terminal geometry using the standard lock-in technique.

### 2.4. Structural Analysis

For structural analysis using TEM, Pd/Bi_2_Se_3_ bilayer samples were prepared following the procedures described in Section 2.2. 

The cross-sectional TEM lamellae of the Pd/Bi_2_Se_3_ bilayer samples were prepared using a Helios G4 focused ion beam (FIB) system manufactured by Thermo Fisher Scientific (Waltham, MA, USA). For protection, about 1 μm of Pt was deposited on top of the bilayer sample before ion milling. After this, a cross-sectional lamella was cut from the Pt-capped sample using FIB and subsequently extracted and transferred onto the FIB-dedicated copper grid using a nanomanipulator for further thinning and polishing. The thinning of the lamella was performed using Ga+ ion beams with an acceleration voltage of 30 kV and a beam current of 230 pA. After thinning, the lamella was polished using Ga+ ions for a few minutes to minimize the surface damage induced by the thinning process. The acceleration voltage and beam current for the polishing process were 2 kV and 23 pA, respectively.

A Scientific Titan Themis Z 60–300 kV electron microscope with condenser lens and objective lens aberration correctors, manufactured by Thermo Fisher Scientific (Waltham, MA, USA), was used to image the atomic arrangement structure of Bi_2_Se_3_ and PdBiSe. The energy-dispersive X-ray spectroscopy (EDX) mappings were gained through a Super-X EDX detector manufactured by Bruker (Billerica, MA, USA). All high-angle annular dark-field imaging (HAADF) images were acquired at an atomic resolution of 80 pm with a beam current of 40 pA, a convergence semiangle of 21.5 mrad, and a collection semiangle snap of 80–379 mrad at 300 kV.

## 3. Results and Discussion

### 3.1. Superconductivity of Pd/Bi_2_Se_3_ Bilayers

The design of the devices (Figure 1a,b) allows two different configurations for four-probe resistance measurements. 

The first configuration uses two independent Pd electrodes as voltage probes, such as electrodes #3 and #4 in device A (Figure 1a). In this configuration, the measured resistance mostly comes from the Bi_2_Se_3_ flake between the Pd electrodes and thus should typically take a finite value. Zero resistance can only be reached when the Bi_2_Se_3_ between the Pd electrodes becomes superconducting.

The second configuration, however, uses two voltage probes that are connected by a Pd “bridge” on top of the Bi_2_Se_3_ flake, such as electrodes #5 and #6 in device A (Figure 1a). The advantage of this configuration is that it allows the detection of the zero-resistance state caused by the superconductivity at the Pd/Bi_2_Se_3_ interface. In the absence of superconductivity, the measured resistance mainly comes from the Pd bridge and thus takes a finite value. However, once superconductivity occurs at the Pd/Bi_2_Se_3_ interface, the two voltage probes will be effectively shorted by the supercurrent. As a result, the measured four-probe resistance will drop to zero.

Surprisingly, at low temperatures, zero-resistance states were detected using both measurement configurations, as shown in Figure 1c,d. According to the discussion above, these observations indicate that a superconducting phase forms not only directly beneath the Pd electrodes but also in the region within a few hundred nanometers from their edges, leading to a superconducting path connecting the two adjacent Pd electrodes. In device A, the R(t) curve measured using independent Pd electrodes exhibits a lower $T_{c}$ compared to that measured across the Pd bridge, as shown in Figure 1c, suggesting that the superconductivity developing between the Pd electrodes is weaker than that beneath the Pd layers. In contrast, in device B, the strength of superconductivity was found to be comparable across different regions in terms of $T_{c}$, which may be attributed to the longer lateral diffusion length of Pd in device B.

**Figure 2:**
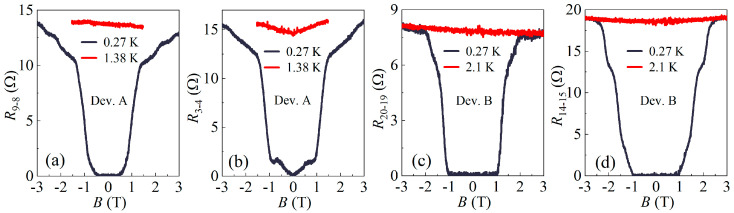
(**a**,**b**) Magnetic field dependence of the four-probe resistance (**a**) *R*_9-8_ and (**b**) *R*_3-4_ of device A, measured at 0.27 K and 1.38 K. (**c**,**d**) Magnetic field dependence of the four-probe resistance (**c**) *R*_20-19_ and (**d**) *R*_14-15_ of device B, measured at 0.33 K and 2.1 K. All curves were measured with an excitation current of 50 nA.

To obtain the upper critical magnetic field $B_{c2}$ of the observed superconducting phase, we measured the R(B) curves of the devices at low temperatures, as plotted in Figure 2. Using 50% of the normal-state resistance as the criterion, the $B_{c2}$ values obtained in different regions of the devices range between 1 T and 1.5 T, all below the Pauli-limiting field estimated using $B_{P}[T]\approx 1.84\;T_{c}[K]$ [16]. This indicates that the main mechanism for the suppression of superconductivity in magnetic fields is the orbital effect. 

In addition, it is noteworthy that the $B_{c2}$ values obtained in the region between independent Pd electrodes are comparable to those obtained beneath the Pd bridge, suggesting that the supercurrents flowing between adjacent Pd electrodes are not due to the Josephson effect but rather originate from bulk superconducting states.

The coherence length of the superconducting states beneath the Pd bridge in device A was estimated to be $\xi (0)=\sqrt{\frac{\Phi_{0}}{2\pi B_{c2}(0)}}\approx 17$ nm, where $\Phi_{0}$ is the flux quantum. The value of $B_{c2}(0)$ was obtained by fitting the Ginzburg–Landau equation to the $B_{c2}(T)$ data, as shown in Figure S1 in the Supplementary Materials. Such a ξ is significantly smaller than both the thickness of the Bi_2_Se_3_ flake and the distance between adjacent Pd electrodes.

### 3.2. Shubnikov–de Haas Oscillations of Bi_2_Se_3_ Flakes

**Figure 3:**
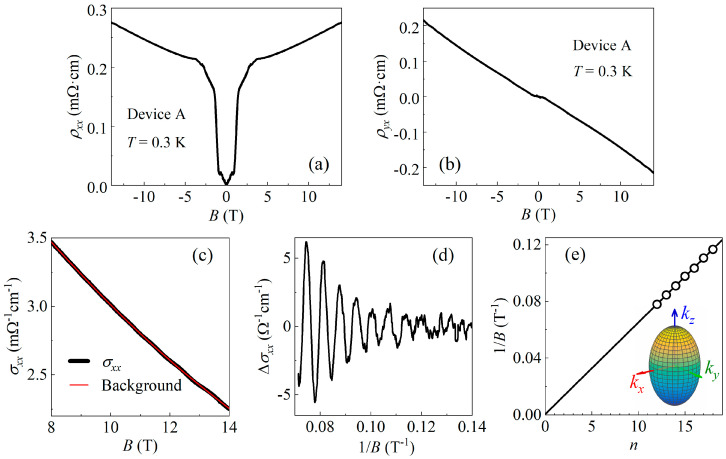
(**a**,**b**) The $\rho_{xx}(B)$ and $\rho_{yx}(B)$ curves of device A, where $\rho_{xx}(B)$ was measured using electrodes #3 and #4, and $\rho_{yx}(B)$ was measured using electrodes #3 and #12. High-field $\sigma_{xx}(B)$ data obtained using $\sigma_{xx}=\rho_{xx}/(\rho_{xx}^{2}+\rho_{yx}^{2})$, showing clear SdH oscillations. (**c**) The background of classical magnetoconductivity was obtained by smoothing the $\sigma_{xx}(B)$ curve, as shown by the red line in the figure. (**d**) The $∆\sigma_{xx}$ data obtained by subtracting the background, plotted against 1/B. (**e**) Landau fan diagram plotted using the minima of oscillations in (**d**). A linear fit to the data gives a zero intercept and a slope of 155 T. Inset: schematic diagram of the elliptical bulk Fermi surface of Bi_2_Se_3_.

Since zero-resistance states were detected between adjacent Pd electrodes, it is important to clarify whether the Bi_2_Se_3_ flake in this region remained intact. To investigate this issue, we measured the magnetoresistivity $\rho_{xx}(B)$ and Hall resistivity $\rho_{yx}(B)$ of the Bi_2_Se_3_ flake in device A using electrodes #3, #4, and #12. The results are plotted in Figure 3a,b.

In high magnetic fields where superconductivity is suppressed, Shubnikov–de Haas (SdH) oscillations were observed in both the $\rho_{xx}(B)$ and $\rho_{yx}(B)$ curves. To obtain more information, the $\sigma_{xx}(B)$ curve was calculated using $\sigma_{xx}=\rho_{xx}/(\rho_{xx}^{2}+\rho_{yx}^{2})$ in the high-field region, as shown in Figure 3c. After subtracting the background of classical magnetoconductivity, clear signals of SdH oscillations were extracted, as plotted as a function of 1/B in Figure 3d.

Figure 3e shows the Landau fan diagram of the SdH oscillations presented in Figure 3d. The horizontal axis of the figure represents the Landau level index n, while the vertical axis represents the inverse of the magnetic field at which the oscillation minima occur. A linear fit to the data yields an oscillation frequency of F=155 T and an intercept close to zero. The obtained frequency is in good agreement with previously reported values in Bi_2_Se_3_ in the literature [17,18,19]. Additionally, the zero intercept is also consistent with the zero Berry phase expected for bulk electrons in Bi_2_Se_3_, suggesting that the observed SdH oscillations originate from the bulk carriers of the Bi_2_Se_3_ flake.

The Fermi surface of Bi_2_Se_3_ is an ellipsoid with $k_{c}/k_{a,b}=1.62$ [20], as illustrated in the inset of Figure 3e. The carrier density of Bi_2_Se_3_ is thus given by

(1) $$n=\frac{1}{3\pi^{2}}\left(\frac{k_{c}}{k_{a,b}}\right){\left(\frac{2eF}{\hbar }\right)}^{\frac{3}{2}}.$$

Substituting F=155 T into Equation (1) leads to a carrier density of $n=1.7\times 10^{19}$ cm^−3^, which is a typical value for bulk carriers of Bi_2_Se_3_ [17]. Together with the resistance value measured at T=2 K, we obtain the bulk mobility of the Bi_2_Se_3_ flake μ=1794 cm^2^V^−1^s^−1^.

The results above indicate that, from the perspective of electron transport, the crystal quality of the Bi_2_Se_3_ flake between the adjacent Pd electrodes is not affected. In the following section, we will further explore the reasons for these results through structural analysis.

### 3.3. Formation of Superconducting PdBiSe

To investigate the origin of the observed superconductivity, atomic-resolution elemental and structural analysis was performed using TEM. The technical details of the TEM measurements are presented in Section 2.4. 

**Figure 4:**
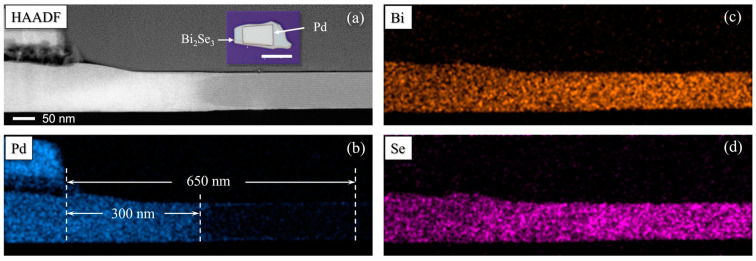
(**a**) Cross-sectional HAADF image of a Pd/Bi_2_Se_3_ bilayer, taken at the edge of the Pd layer. Inset: optical photo of an as-fabricated Pd/Bi_2_Se_3_ bilayer sample, with a scale bar of 10 μm. (**b**–**d**) EDX elemental maps of (**b**) Pd, (**c**) Bi, and (**d**) Se of the Pd/Bi_2_Se_3_ bilayer.

Figure 4a shows a cross-sectional HAADF image taken near the edge of the Pd layer. The EDX elemental maps of this region (Figure 4b–d) provide clear evidence of Pd diffusion from the top Pd layer into the Bi_2_Se_3_ layer underneath. As shown in Figure 4b, Pd fully penetrates through the Bi_2_Se_3_ flake in the regions directly beneath the Pd layer and within about 300 nm from the edge. Meanwhile, in areas about 300 nm to 650 nm away from the edge of the Pd layer, Pd continues to diffuse outward along the top and bottom surfaces of the Bi_2_Se_3_ flake, but no distribution of Pd is observed in the center of the Bi_2_Se_3_ flake.

**Figure 5:**
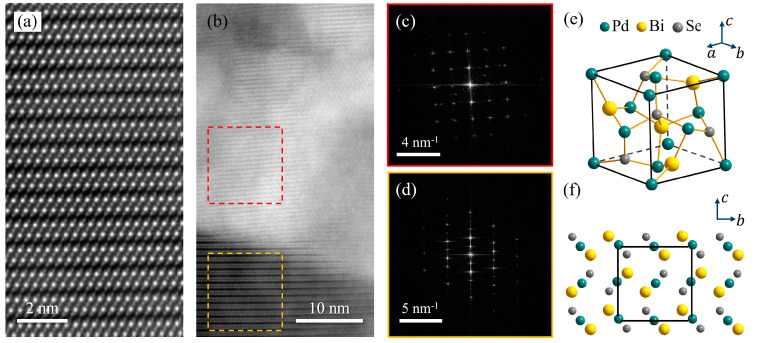
(**a**) Atomic-resolution HAADF image of the Bi_2_Se_3_ layer far from the Pd/Bi_2_Se_3_ interface, showing the clear stacking of Se-Bi-Se-Bi-Se quintuple layers. (**b**) HAADF image at the Pd/Bi_2_Se_3_ interface. A crystalline phase was found to form in the Pd-rich area. (**c**,**d**) 2D FFT patterns of the areas indicated by the dashed (**c**) red and (**d**) yellow boxes in (**b**). The lattice constant extracted from the data in (**c**) is 0.63 nm, fairly close to that of cubic PdBiSe. (**e**) The unit cell of PdBiSe, with a lattice constant of 0.64 nm. (**f**) Schematics of the PdBiSe crystal structure. The black box represents the unit cell.

To investigate whether the observed superconductivity in the Bi_2_Se_3_/Pd bilayers was caused by Pd diffusion, we imaged both the Pd-free and Pd-rich regions in the Bi_2_Se_3_ flake using TEM. In Pd-free regions far from the Pd/Bi_2_Se_3_ interface, the crystal structure of Bi_2_Se_3_ remains intact, exhibiting the clear stacking of Se-Bi-Se-Bi-Se quintuple layers, as shown in Figure 5a. However, in Pd-rich regions, the sample exhibits a crystal structure distinctly different from that of Bi_2_Se_3_, as shown in the region enclosed by the red dashed box in Figure 5b.

The difference in the lattice structures of these two regions is also evident in their 2D fast Fourier transform (FFT) patterns, as shown in Figure 5c,d. The square-shaped FFT pattern of the Pd-rich region (Figure 5c) indicates that the crystalline compound found in this area has a cubic lattice structure, with a lattice constant of 0.63 nm. After a comprehensive comparison of the crystal structure and lattice constant with those of known materials in the database, the observed compound was identified as PdBiSe. 

PdBiSe is a superconductor with a noncentrosymmetric cubic structure, as illustrated in Figure 5e,f. The lattice constant of PdBiSe is 0.64 nm, which is very close to that of the crystalline compound found in the Pd-rich regions of the Pd/Bi_2_Se_3_ bilayers [21]. Although PdBiSe is not a van der Waals material, it exhibits a layered structure when viewed along the a-axis, as illustrated in Figure 5f. Such a layered structure is consistent with the layer-like patterns observed in the Pd-rich region in Figure 5b.

The bulk $T_{c}$ of PdBiSe is approximately 1.5 K, slightly higher than the maximum $T_{c}$ of 1.25 K observed in our devices (Figure 1c). Since the $T_{c}$ of a superconductor is often influenced by various factors, such as sample size and crystal quality, such a small difference in $T_{c}$ is not surprising.

## 4. Further Discussion

In our early work, we (F.Y., G.L., and L.L) used Pd electrodes as normal-metal contacts to probe the proximity-induced superconductivity in Bi_2_Se_3_ [22], unaware that a superconducting compound could form at the Pd/Bi_2_Se_3_ interface. With the knowledge gained from this study, the reinterpretation of the results in ref. [22] is necessary.

## 5. Conclusions

We discovered that sputtering Pd onto Bi_2_Se_3_ flakes leads to the formation of a crystalline PdBiSe layer with a superconducting transition temperature of $T_{c}\approx$ 1.2 K. These findings not only deepen the understanding of the physical and chemical processes at the Pd/Bi_2_Se_3_ interface but also provide a practical approach for the introduction of superconductivity into the topological insulator Bi_2_Se_3_, and therefore will be helpful for the development of Bi_2_Se_3_-based hybrid superconducting devices. 

## Data Availability

The data presented in this study are available on request from the corresponding authors (due to privacy).

## Supplementary Materials

The following supporting information can be downloaded at: https://www.mdpi.com/article/10.3390/ma17225460/s1, Figure S1: The $R_{9–8}(B)$ curves measured at various temperatures and the $B_{c2}(T)$ data extracted from the $R_{9–8}(B)$ curves.
