# Supplementary material for: Superconductivity at Pd/Bi2Se3 Interfaces Due to Self-Formed PdBiSe Interlayers
Source: Materials (Basel). 2024 Nov 8;17(22):5460. doi: 10.3390/ma17225460 (PMC11595955; doi:10.3390/ma17225460)
Supplement: Supplementary file 1 [file materials-17-05460-s001.zip › materials-3300966-supplementary.pdf]

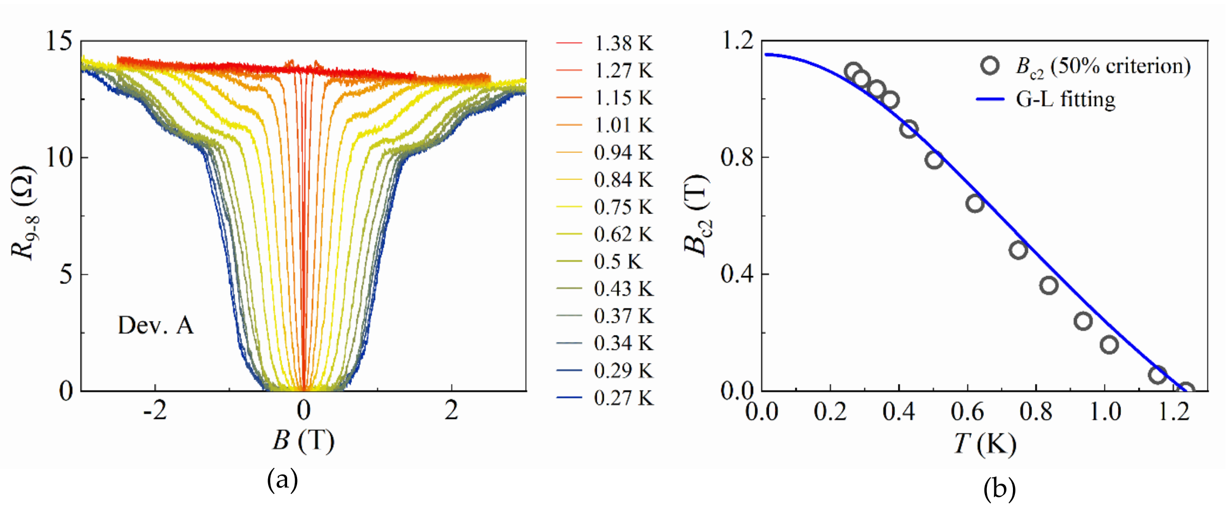

Figure S1. (a) The $R_{9.8}(B)$ curves of device A, measured at various temperatures. (b) The $B_{c2}(T)$ data extracted from the curves in (a). Here, the B_{c2} is defined as the magnetic field at which $R_{9.8}$ reaches 50% of its normal-state value. The blue line represents the best fit to the $B_{c2}(T)$ data using the Ginzburg-Landau equation $B_{c2}(T) = B_{c2}(0)\left[\frac{1-(T/T_c)^2}{1+(T/T_c)^2}\right]$, which yields $B_{c2}(0) = 1.15$ T.
